# Supplementary material for: MetaRibo-Seq measures translation in microbiomes
Source: Nat Commun. 2020 Jun 29;11:3268. doi: 10.1038/s41467-020-17081-z (PMC7324362; doi:10.1038/s41467-020-17081-z)
Supplement: Supplementary file 10 — Supplementary Data 7 [file 41467_2020_17081_MOESM10_ESM.zip › File2/Confidence_High_Taxonomy/296343_out.krona.html]

Javascript must be enabled to view this page.

members
magnitude
magnitudeUnassigned
count
unassigned
taxon
rank

296343\_out

2

2
2
superkingdom

1
1239
phylum

1
186801
class

1
186802
order

31979
1
family

genus
1
1485

species

SRS019693\_contig\_number\_3556
1
1896978

1
95818
phylum

species

SRS024355\_contig\_number\_contig-100\_8987.118711
1
447454
